# The neurodegenerative diseases ALS and SMA are linked at the molecular level via the ASC-1 complex

**DOI:** 10.1093/nar/gky1093

**Published:** 2018-11-06

**Authors:** Binkai Chi, Jeremy D O’Connell, Alexander D Iocolano, Jordan A Coady, Yong Yu, Jaya Gangopadhyay, Steven P Gygi, Robin Reed

**Affiliations:** Department of Cell Biology, Harvard Medical School, 240 Longwood Ave. Boston MA 02115, USA

## Abstract

Understanding the molecular pathways disrupted in motor neuron diseases is urgently needed. Here, we employed CRISPR knockout (KO) to investigate the functions of four ALS-causative RNA/DNA binding proteins (FUS, EWSR1, TAF15 and MATR3) within the RNAP II/U1 snRNP machinery. We found that each of these structurally related proteins has distinct roles with FUS KO resulting in loss of U1 snRNP and the SMN complex, EWSR1 KO causing dissociation of the tRNA ligase complex, and TAF15 KO resulting in loss of transcription factors P-TEFb and TFIIF. However, all four ALS-causative proteins are required for association of the ASC-1 transcriptional co-activator complex with the RNAP II/U1 snRNP machinery. Remarkably, mutations in the ASC-1 complex are known to cause a severe form of Spinal Muscular Atrophy (SMA), and we show that an SMA-causative mutation in an ASC-1 component or an ALS-causative mutation in FUS disrupts association between the ASC-1 complex and the RNAP II/U1 snRNP machinery. We conclude that ALS and SMA are more intimately tied to one another than previously thought, being linked via the ASC-1 complex.

## INTRODUCTION

Understanding the mechanisms that underlie pathogenesis of the fatal neurodegenerative disease Amyotrophic Lateral Sclerosis (ALS) is an area of intense investigation. Greater than 25 genes have been identified as causes for ALS (1). These genes have roles in a wide variety of processes, including gene expression, mitochondrial function, protein degradation, autophagy, apoptosis, and nuclear-cytoplasmic transport (2–6). At present, the contribution of each process to ALS pathogenesis is not known.

Many of the ALS-causative genes encode RNA/DNA binding proteins (7,8). The best known of these are Fused in Sarcoma (FUS) and TAR DNA Binding Protein (TARDBP) (9–13). FUS is one of the three members of the structurally related FET (FUS, EWSR1 and TAF15) family of RNA/DNA binding proteins (14). In addition to the RNA/DNA binding domains, the FET proteins also contain low-complexity domains, and these domains are thought to be involved in ALS pathogenesis (5,15). In light of the discovery that mutations in FUS are ALS-causative, several groups carried out studies to determine whether the other two members of the FET family, TATA-Box Binding Protein Associated Factor 15 (TAF15) and EWS RNA Binding Protein 1 (EWSR1), have a role in ALS. At present, the case is strongest for TAF15, but evidence is accumulating that mutations in EWSR1 are also ALS-causative (7,16–20). More recently, ALS-causative mutations were found in Matrin 3 (MATR3), which similar to the FET family, contains RNA/DNA binding motifs as well as low-complexity domains (21). As has been established for the vast majority of ALS-causative genes, the mode of inheritance for FUS and MATR3 is dominant (21,22). Further studies are needed for TAF15 and EWSR1. In light of the evidence that the FET family members and MATR3 are associated with ALS and share structural and biochemical similarities, we will refer to all four proteins in our study as ALS-causative for simplicity. Multiple studies have shown that the four ALS-causative proteins have numerous functions, including transcription, splicing, mRNA export, the DNA damage response and formation of membraneless organelles (7,8,14,23). It is not yet known how these functions and disruption of these functions relate to ALS pathogenesis.

In a study that was done concurrently with the work presented below, we characterized the interactomes of FUS, EWSR1, TAF15 and MATR3 (24). This analysis revealed multiple unique interactors for each ALS-causative protein and identified U1 small nuclear ribonucleoprotein particle (U1 snRNP) as a common factor to all four interactomes. This essential splicing factor, which recognizes 5′ splice sites in introns, functions at the earliest steps of spliceosome assembly (25–29). The observation that U1 snRNP associates with all of the ALS-causative proteins in our study led us to characterize the U1 snRNP interactome in detail. Unexpectedly, we found that the components of immunopurified U1 snRNP overlapped extensively with those of immunopurified RNA Polymerase II (RNAP II). We had initially characterized this essential transcription machinery more than a decade ago (30). In the study below, we present an up-to-date analysis of immunopurified RNAP II, which further reveals its extensive overlap with immunopurified U1 snRNP. Thus, based on our recent work (24) and the present study, we now refer to immunopurified RNAP II as the RNAP II/U1 snRNP machinery (see Results for details). We are especially interested in this machinery because our data reveal that it houses >1/3 of all known ALS-causative proteins. Remarkably, it also contains 5 proteins that are Spinal Muscular Atrophy (SMA)-causative. Thus, the pathways in which the RNAP II/U1 snRNP machinery function are highly germane to these motor neuron diseases. In order to gain insight into the pathways, we have now focused our attention on understanding the roles of the ALS-causative proteins FUS, EWSR1, TAF15 and MATR3 in this machinery. To do this we carried out CRISPR knock out (KO) of each gene in HeLa cells and examined the effects on the RNAP II/U1 snRNP machinery. Notably, this analysis revealed that all four ALS-causative proteins are required for association of the RNAP II/U1 snRNP machinery with a transcriptional co-activator known as the Activating Signal Cointegrator 1 (ASC-1) complex (31). This result is of special significance as mutations in ASC-1 components are known to cause SMA (32,33). Previously, we and others found that FUS directly interacts with Survival of Motor Neuron 1 (SMN1), the main cause of SMA, providing the first evidence that SMA and ALS are linked at the molecular level (34–38). Our new result with the ASC-1 complex is now the second reported molecular link between ALS and SMA. The observation that the ASC-1 complex, FUS, SMN1 and numerous other ALS/SMA-causative proteins are present in the RNAP II/U1 snRNP machinery underscores the central importance of this machinery to motor neuron disease.

## MATERIALS AND METHODS

### Plasmids and antibodies

The hCas9 plasmid was a gift from George Church (Addgene plasmid # 41815). The GST-FUS plasmid was constructed by inserting the coding sequence of FUS into the BamHI and XhoI sites of pGEX-6P-1 (34). The FUS^G156E^ or FUS^R514G^ mutation was introduced into the GST-FUS plasmid using the QuikChange II Site-Directed Mutagenesis Kit (Agilent, Santa Clara, CA, USA). The GST-DExD-Box Helicase 39B (DDX39B) plasmid was a generous gift from Michael R. Green (39). The HA-TRIP4 plasmid was constructed by inserting the coding sequence of TRIP4 into the KpnI and BamHI sites of pcDNA3.1+N-HA using Clone EZ technology (GenScript, Piscataway, NJ, USA). The HA-TRIP4^1-254^ plasmid was generated by mutagenesis using the QuikChange II Site-Directed Mutagenesis Kit (Agilent) and the HA-TRIP4 plasmid as template. The monoclonal antibodies used in this study were 8WG16 (against RNA Polymerase II Subunit A (POLR2A), which is the large subunit of RNAP II) from Biolegend (San Diego, CA) (cat # 920102), Small Nuclear Ribonucleoprotein Polypeptide C (SNRPC) from Sigma-Aldrich (St. Louis, MO, USA) (cat # SAB4200188) and Lamin A/C (LMNA) (cat # sc-7293) from Santa Cruz (Dallas, TX, USA). Polyclonal antibodies against EWSR1 (cat # A300-418A), MATR3 (cat # A300-591A), HA (cat # A190-108A), ASCC1 (cat # A303-871A), ASCC2 (cat # A304-020A), ASCC3 (cat # A304-014A) and TRIP4 (cat # A300-203A) were from Bethyl (Montgomery, TX, USA), and the polyclonal antibody against TAF15 was from Novus (Littleton, CO) (cat # NB100-567). We used our rabbit polyclonal antibodies to FUS (34) and EIF4A3 (40).

### Immunoprecipitations (IPs)

For IPs, antibodies were coupled to Protein A Sepharose beads (GE healthcare, Marlborough, MA, USA) at a 1:1 ratio of beads to antibody and covalently cross-linked to the beads using dimethylpimelimidate (Sigma-Aldrich) as described (41). Reaction mixtures (1 ml) containing 300 μl of HeLa nuclear extract (42), 300 μl of splicing dilution buffer (20 mM HEPES, pH 7.9, 100 mM KCl), 500 μM ATP, 3.2 mM MgCl_2_ and 20 mM creatine phosphate (di-Tris salt) were incubated for 20 min at 30°C. This incubation turns over endogenous complexes in the nuclear extract (30). Reaction mixtures were then added to 500 μl of buffer A (1× PBS, 0.1% Triton, 0.2 mM PMSF, protease inhibitor EDTA-free [Roche, Basel, Switzerland]) and 40 μl of antibody-crosslinked beads. The mixture was rotated overnight at 4°C, followed by five washes with buffer A. Proteins were eluted at room temperature with 80 μl of protein gel loading buffer lacking DTT (125 mM Tris pH 6.8, 5% SDS, 20% glycerol, 0.005% bromophenol blue). After elution, DTT was added to a final concentration of 40 mM, and 15 μl of each eluate was separated on a 4–12% SDS-PAGE gradient gel (Life technologies, Carlsbad, CA, USA). All IPs were carried out in the absence of RNase A, except for Figure 2 and Supplementary Figure S6. For the RNase-treated IPs, RNase A (50 ng/μl, Promega, Madison, WI, USA) was added to the IP reaction mixture prior to the incubation at 30°C for 20 min. The remaining IP steps were carried out with the same methods used for IPs without RNase treatment.

### Complementation of KO extracts with recombinant proteins

For add back experiments, recombinant proteins (GST-DDX38B, GST-FUS, GST-FUS^G156E^ and GST-FUS^R514G^) were expressed in *Escherichia coli* and affinity purified using Glutathione Sepharose 4B resin (GE Healthcare). Purified proteins were added to the KO extracts prior to the incubation at 30°C for 20 min. We used the amount of recombinant proteins that was same as the amount of endogenous FUS in the KO extract as determined by Western analysis. The remaining IP steps were carried out with the same methods used for IPs without add back.

### Association of WT and mutant TRIP4 with RNAP II

To assay for association of WT or SMA-causative TRIP4 mutant (TRIP4^1–254^) with RNAP II, transfection assays were carried out. To do this, HA-TRIP4, HA-TRIP4^1–254^ or HA-DDX39B (as a negative control) were transfected into HeLa cells. After 24 h, cells were harvested and whole cell lysates were prepared from 1 × 10^7^ cells transfected with the plasmids. Cells were resuspended in 500 μl of buffer A and lysates were prepared by sonication for 12 s followed by centrifugation at 12 000 rpm at 4°C for 10 min. The whole cell lysate supernatants were then used for IPs with RNAP II or EIF4A3 (as a negative control) antibodies. This was performed by rotating cell lysates at 4°C overnight together with 40 μl of antibody-crosslinked beads. The washing and elution steps were carried out under the same conditions used for IPs from nuclear extract.

### Mass spectrometry

For quantitative mass spectrometry of immunopurified RNAP II in wild type or KO ALS lines, the immunoprecipitates were Trichloro Acetic Acid (TCA) precipitated and the digested peptides were labeled by tandem mass tag (43) for MS3 analysis on an Orbitrap Fusion mass spectrometer coupled to a Proxeon EASY-nLC 1000 liquid chromatography (LC) pump (Thermo Scientific, Waltham, MA, USA). The proteins in Supplementary Tables S1, S3 and S4 were annotated with functions using the Gene Cards database (www.genecards.org) (44) and/or searching the literature. Abundant cytoplasmic proteins, ribosomal proteins, proteins greater than 200 kDa with <10 spectral counts, proteins >70 kDa with less than four spectral counts, and proteins with one spectral count were not included. The amounts of the proteins were normalized to the amount of POLR2A in each extract. Fold change was calculated by comparing the amount of each protein in immunopurified RNAP II from KO extract with that from wild type HeLa extract.

### CRISPR/Cas9 KO cell lines

Guide RNAs were cloned into the pSTBlue-1 vector and co-transfected into HeLa cells together with hCas9 plasmid. After 48 h, single cells were sorted into 96-well plates by Fluorescence Activated Cell Sorting (FACS). Positive clones were identified by Westerns and DNA sequencing. The sequences of the guide RNAs were: 5′-GCGCCCTTACCTACCGTTTG-3′ (FUS), 5′-GGAAGTTACGGTCAGTCTGG-3′ (TAF15), 5′-AGGCAGGCCTTACCAGTGGG-3′ (EWSR1) and 5′-GAGATGGCAGATCTGCTACA-3′ (MATR3).

## RESULTS

### The RNAP II/U1 snRNP machinery

**Figure 1.**
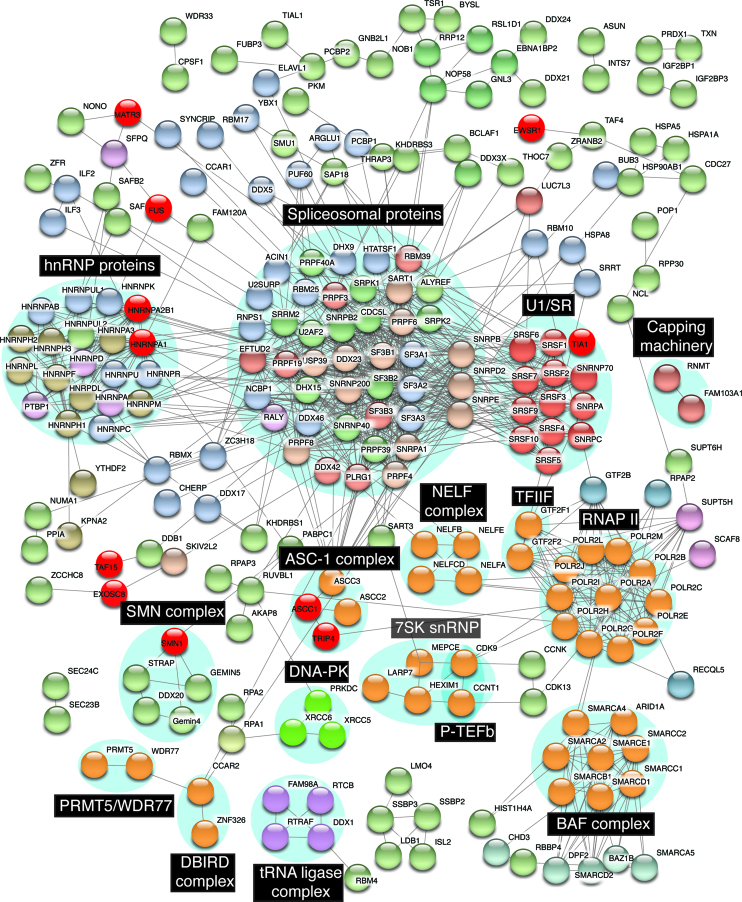
Protein interaction network of the RNAP II/U1 snRNP machinery. The interaction network of the RNAP II/U1 snRNP machinery was built using the STRING database (confidence > 0.7 based on experimental data) together with manual changes as described in the main text. Distinct complexes are labeled in the black boxes. ALS/SMA-causative proteins are marked in bright red.

Previously, we characterized the RNAP II/U1 snRNP machinery immunopurified from HeLa cell nuclear extracts and identified ∼100 proteins components within it (30). Among these were the RNAP II core subunits, numerous transcription factors, RNA/DNA binding proteins, and all of the canonical U1 snRNP components. The FET family proteins (FUS, EWSR1, TAF15) and MATR3 were also detected. Subsequently, these four proteins were reported to be ALS-causative (9,17,18,21). Because of the associations between the ALS-causative proteins and the RNAP II/U1 snRNP machinery, we characterized this machinery in detail using currently available quantitative mass spectrometry technology. We identified 274 proteins, which are listed together with their best-known functions and/or the functions that are relevant to motor neuron disease in Supplementary Table S1. To identify interactions among the components, we used the String database (https://string-db.org, version 10.5) (45). This analysis revealed numerous distinct complexes, many of which were separated into individual clusters by String (Supplementary Figure S1). We note that numerous well-known complexes were not clustered by STRING because the proteins interact with multiple complexes within the RNAP II/U1 snRNP machinery. The STRING algorithm does not cluster the complexes at all when this occurs. Thus, we manually organized the network to generate clusters containing known complexes based on the information we gathered for Supplementary Table S1. In addition, for simplicity, the proteins that were not linked to any other proteins were omitted. Together, these enhancements resulted in the network shown in Figure 1. Among the complexes are several transcription factors, including the Positive Transcription Elongation Factor (P-TEFb) (46), the BRG1/BRM-Associated Factor (BAF) chromatin remodeling complex (47), the negative elongation factor (NELF) complex (48), the ASC-1 complex (31,49) and the transcription factor IIF (TFIIF) (50). We also identified several other complexes. One of these, Protein Arginine Methyltransferase 5/WD Repeat Domain 77 (PRMT5/WDR77) complex, methylates the POLR2A subunit of RNAP II to recruit SMN1 to RNAP II (51). Another complex is known as the Deleted in Breast Cancer 1 (DBC1) and Zinc Finger Protein 326 (ZNF326) complex (DBIRD complex), which integrates alternative splicing with RNAP II transcript elongation (52). We also detected the tRNA ligase complex. In addition to functioning in tRNA splicing (53,54), this complex has a role in RNA transport between the nucleus and cytoplasm in an RNAP II transcription-dependent manner (55). The DNA-dependent protein kinase (DNA-PK) complex that functions in DNA repair (56) was also present (Figure 1). In addition, the core components of U1 snRNP (SNRNP70, SNRPA and SNRPC), and its associated Serine and Arginine Rich Splicing Factor (SRSF) family are present (U1/SR in Figure 1), consistent with our previous work (30). Unlike U1/SR, many of the core components of the other snRNPs are missing. These data indicate that U1/SR is the main snRNP associated with the RNAP II/U1 snRNP machinery. In line with this result, U1 snRNA, but not the other snRNAs, was detected in this machinery (Supplementary Figure S2). As mentioned in the Introduction, we recently characterized immunopurified U1 snRNP and found that its components largely overlap with those of immunopurified RNAP II (Supplementary Table S2 and (24)). Specifically, of the 296 components identified in the two machineries combined, 204 components are shared, 22 are unique to U1 snRNP, and 70 are unique to RNAP II (Supplementary Table S2). We did not detect RNAP II core subunits in immunopurified U1 snRNP, possibly due to a buried epitope. Consistent with this possibility, GST-SRSF1, which pulls down U1 snRNP also pulls down RNAP II, supporting an association between these two machineries (30). Based on these observations, we have defined the 274 components of immunopurified RNAP II as the RNAP II/U1 snRNP machinery.

### ALS/SMA-causative proteins associate with the RNAP II/U1 snRNP machinery

Interestingly, our data indicate that the RNAP II/U1 snRNP machinery contains most of the known ALS-causative RNA/DNA binding proteins (Table 1 and (24)). Strikingly, numerous SMA-causative proteins are also components of the RNAP II/U1 snRNP machinery. These include the Exosome Component 8 (EXOSC8) (57) and the Heat Shock Protein Family B (Small) member 1(HSPB1) (58,59) (Table 1). Mutations in HSPB1 have also been identified in patients with another neuropathy, Charcot-Marie-Tooth disease (58,60). In addition, mutations in Thyroid Hormone Receptor Interactor 4 (TRIP4) and Activating Signal Cointegrator 1 Complex 1 (ASCC1), which are components of the RNAP II/U1 snRNP machinery, are causes of a prenatal form of SMA (Table 1) (32,33). Finally, SMN1 associates with RNAP II/U1 snRNP (Table 1). Together, these data reveal an extensive association between the RNAP II/U1 snRNP machinery and motor neuron disease-causative proteins.

**Table 1.** ALS- and SMA-causative proteins in the RNAP II/U1 snRNP machinery

ALS causative	SMA causative
EWSR1	ASCC1
FUS	EXOSC8
HNRNPA1	HSPB1
HNRNPA2B1	SMN1
MATR3	TRIP4
TAF15	
TARDBP	
TIA1	
VCP	

Several of the motor neuron disease-causative proteins are also present in multi-subunit complexes and/or interact with other factors in the RNAP II/U1 snRNP machinery (color coded red in Figure 1). For example, SMN1 is a component of the SMN complex, and TIA1 Cytotoxic Granule Associated RNA Binding Protein (TIA1) associates with U1 snRNP. In addition, ASCC1 and TRIP4 are components of the ASC-1 transcriptional coactivator complex (Figure 1). Importantly, the presence of these proteins in discrete complexes means that the other factors in the complexes are candidates for causing or affecting motor neuron disease course. This point is best exemplified by the ASC-1 complex, in which case mutations in two components of this complex cause SMA.

### Distinct functions for ALS proteins in the RNAP II/U1 snRNP machinery

**Figure 2.**
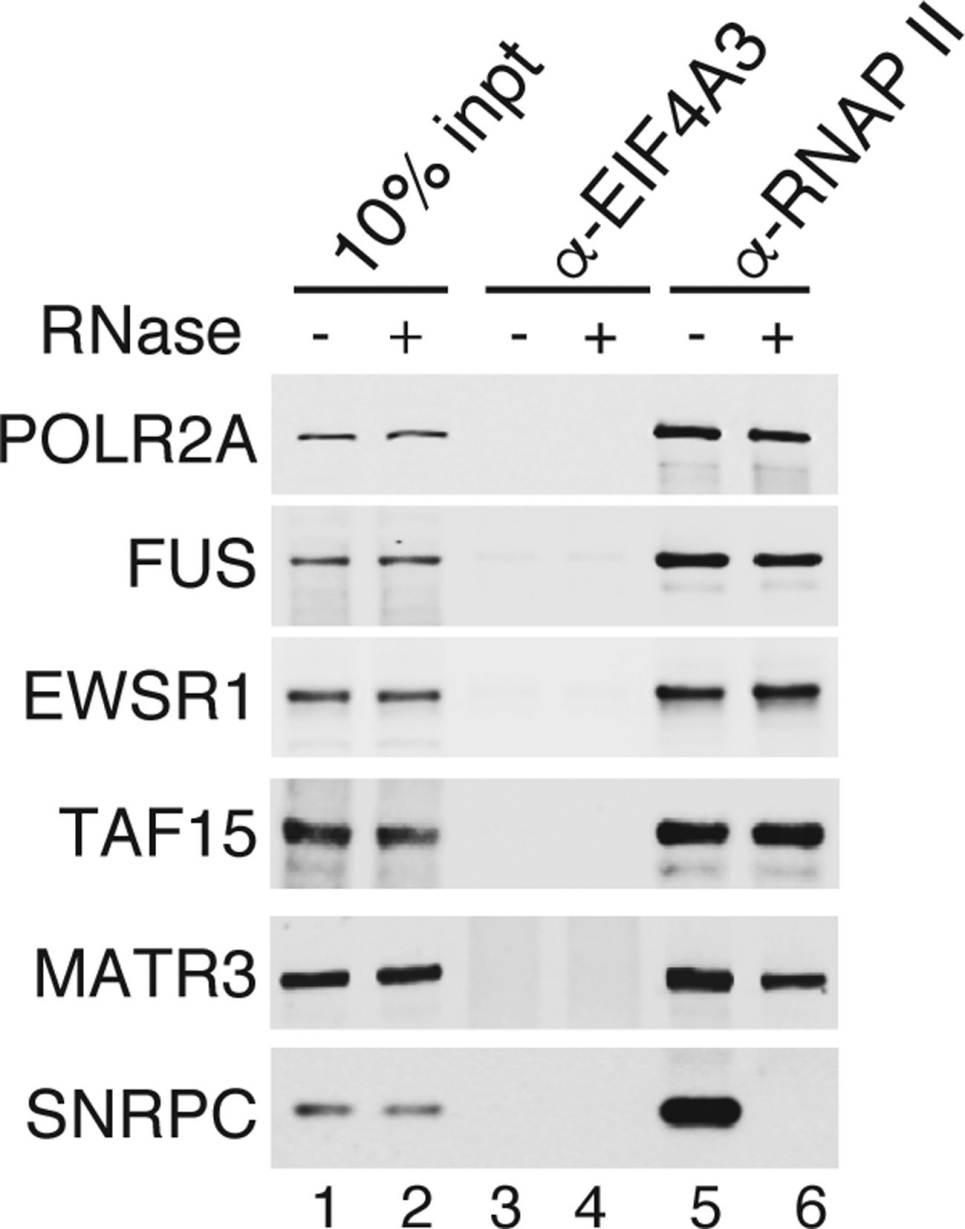
ALS proteins associate with the RNAP II/U1 snRNP machinery in an RNA-independent manner. IPs were carried out from RNase A-treated or untreated nuclear extract using an RNAP II or a negative control antibody (EIF4A3) followed by westerns with the indicated antibodies.

We next investigated the roles of FUS, TAF15, EWSR1 and MATR3 (hereafter referred to as ALS proteins for brevity) in the RNAP II/U1 snRNP machinery. Previously, we showed that these proteins all co-IP with U1 snRNP (24). As shown in Figure 2, they also co-IP with RNAP II. These data validate their association with the RNAP II/U1 snRNP machinery. All of the ALS proteins, but not the U1 snRNP component SNRPC, co-IP with RNAP II in the presence of RNase, indicating that U1 snRNP or other RNAs do not mediate interactions between the ALS proteins and the RNAP II/U1 snRNP machinery.

**Figure 3.**
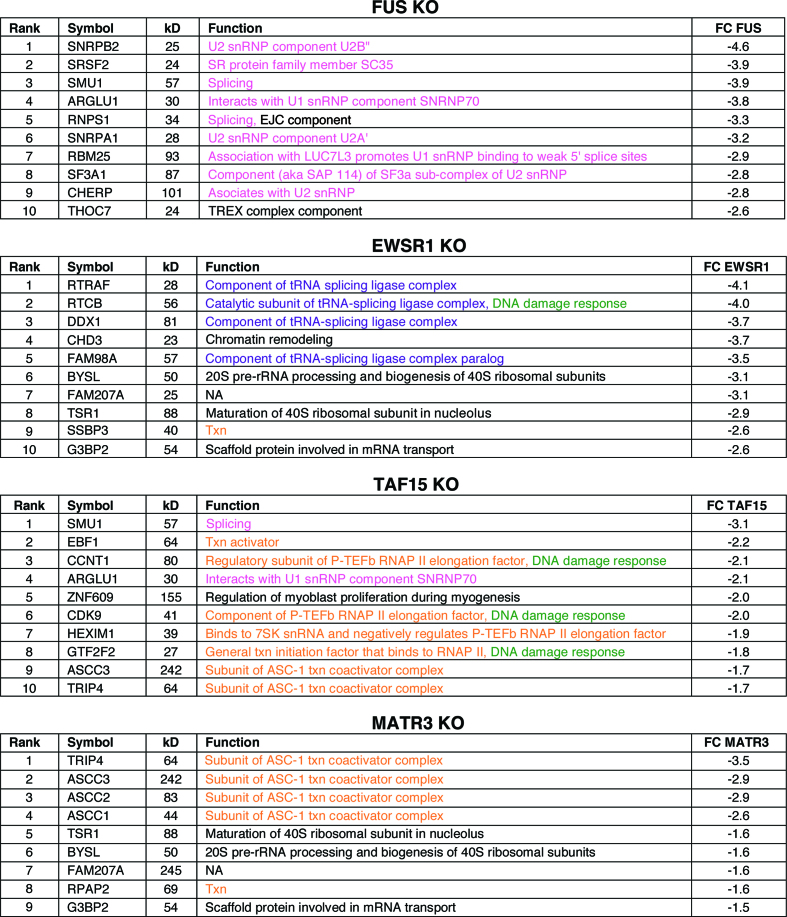
Top hit proteins dissociated from the RNAP II/U1 snRNP machinery in ALS protein KOs. The top ranked (by fold change) proteins that dissociated from the RNAP II/U1 snRNP machinery in each KO line are shown. The rank, HGNC official symbol, calculated molecular weight (kD), best-known function, and fold change relative to wild type are shown. Functions in splicing (pink), transcription (txn, orange), DNA damage response (green), tRNA splicing ligase complex (purple) and other (black) are indicated.

To further examine the roles of the ALS proteins in the RNAP II/U1 snRNP machinery, we generated CRISPR KO HeLa cell lines for each protein. As shown in Supplementary Figure S3, the target protein was specifically knocked out. We then immunopurified the RNAP II/U1 snRNP machinery from ALS protein-KO nuclear extracts and compared it to the wild type machinery. The changes in levels of each component normalized against POLR2A are shown in Supplementary Table S3. A low level of each ALS protein is detected in its respective KO line, which is a known consequence of instrumental noise and/or ion interference (61). We then focused on the proteins with the most significant negative (≤–1.5) fold change. The levels of the core subunits of RNAP II were not changed in the KO lines (Supplementary Figure S4). The top hits that dissociate from the RNAP II/U1 snRNP machinery in the different KO lines are shown in Figure 3, and the full lists are shown in Supplementary Table S4. In the FUS KO, the majority of proteins that dissociate are splicing factors (color coded pink, Figure 3 and Supplementary Supplementary Table S4). In the EWSR1 KO, four out of the top five hits are components of the tRNA ligase complex (color coded purple, Figure 3) whereas most of the top hits in the TAF15 KO are transcription factors (color coded orange, Figure 3). Together, these data indicate that, despite the similarity of the three FET family members, they largely play distinct roles within the RNAP II/U1 snRNP machinery. Finally, the top hits in the MATR3 KO are components of the ASC-1 complex (color coded orange, Figure 3, and see below).

As described above and shown in Figure 1, we identified multiple complexes in the RNAP II/U1 snRNP machinery. To further investigate the presence of these complexes in the machinery, we separated total nuclear extract by gel filtration and then carried out IPs from the relevant fractions followed by Westerns with antibodies against multiple components of the complexes identified in Figure 1. This analysis validated the presence of all of the components examined, including subunits of the tRNA ligase complex, NELF, P-TEFb, and the ASC-1 complex (Supplementary Figure S5).

**Figure 4.**
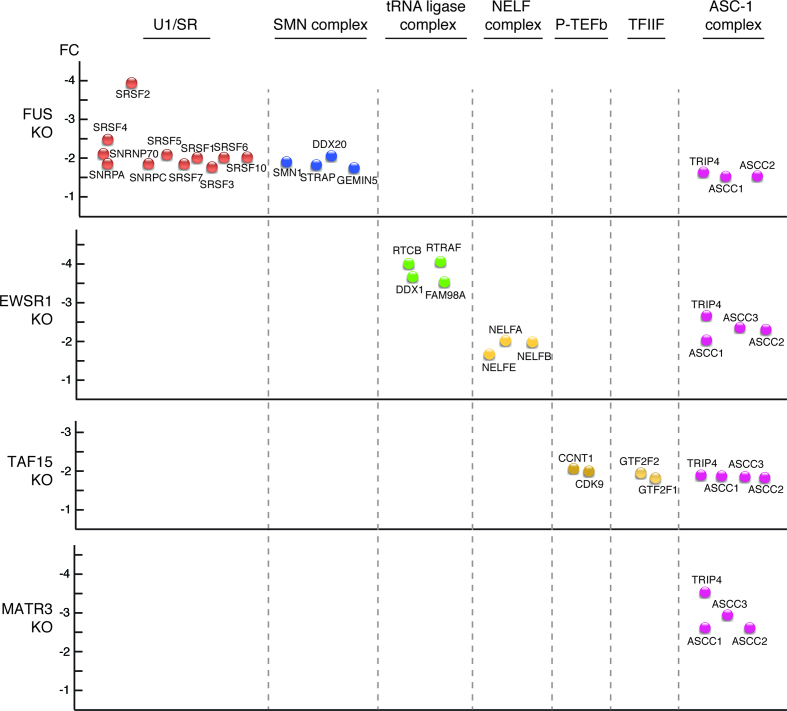
ALS protein KOs disrupt association of distinct complexes with the RNAP II/U1 snRNP machinery. Protein complexes that dissociate from the RNAP II/U1 snRNP machinery in FUS, EWSR1, TAF15 or MATR3 KO lines are shown. The Y axis indicates fold change (FC).

We next examined whether association of these complexes with the machinery was affected by the different KOs (Figure 4). Consistent with our previous work showing that FUS mediates an association between U1/SR and RNAP II (62), U1/SR dissociates in the FUS KO, and this role is unique to FUS (Figure 4). The SMN complex also dissociated from the RNAP II/U1 snRNP machinery only in the FUS KO, in line with the observation that FUS associates with the SMN complex (34). The tRNA ligase complex and NELF both dissociated in the EWSR1 KO, effects specific to EWSR1. Transcription factors P-TEFb and TFIIF only dissociated in the TAF15 KO. Our data indicate that each of the complexes dissociate as a whole from the RNAP II/U1 snRNP machinery, rather than as individual components, because the fold changes are similar for the components of each complex (Figure 4). Thus, dissociation of these complexes is likely to be physiologically relevant rather than non-specific dissociation of individual proteins.

Interestingly, the only common effect of all four KOs was dissociation of the ASC-1 complex from the RNAP II/U1 snRNP machinery (Figure 4). In light of the observation that mutations in two of its components (ASCC1 and TRIP4) are SMA causative (32,33), we further pursued the relationship between the ASC-1 complex and the ALS proteins. We first carried out IP/Westerns to validate the association of the ASC-1 complex with the RNAP II/U1 snRNP machinery. As shown in Supplementary Figure S6, all four components of the ASC-1 complex co-IP with RNAP II. In addition, this association is RNase-resistant (Supplementary Figure S6), indicating that protein-protein interactions are involved.

**Figure 5.**
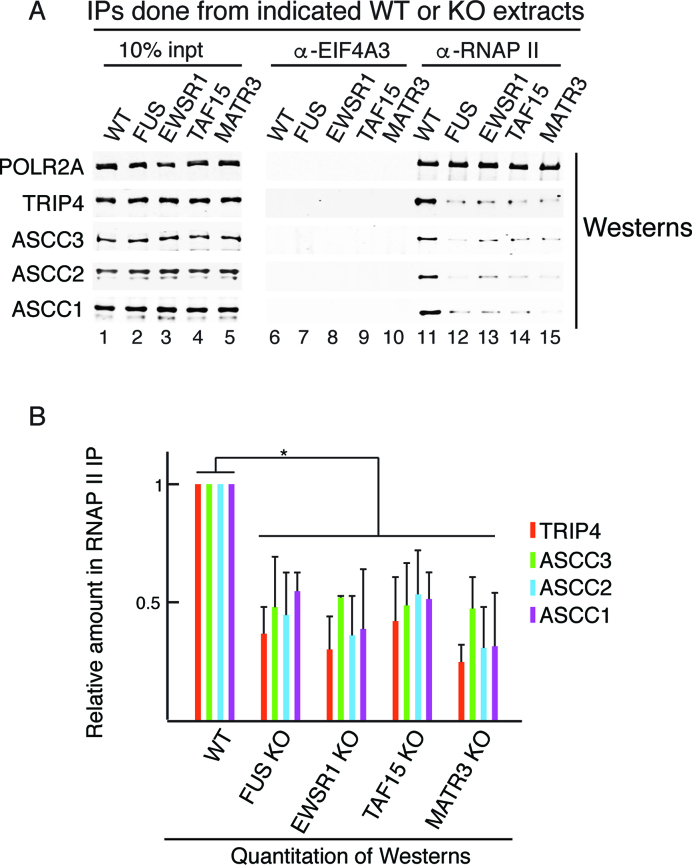
All four ALS proteins mediate association of the ASC-1 complex with the RNAP II/U1 snRNP machinery. (**A**) IP/Western analysis of ASC-1 components associated with the RNAP II/U1 snRNP machinery in WT or KO lines. (**B**) Three independent replicates of the IP/Westerns shown in (A) were quantitated. The bars indicate the mean values of fold change. Error bars represent standard deviations. *P < 0.05 (two-tailed student's *t*-test).

We next carried out IP/Westerns to examine the association of the ASC-1 components with the RNAP II/U1 snRNP machinery in the four ALS proteins KOs (Figure 5A). Quantitation of these data is shown in Figure 5B, which confirms that the ASC-1 complex dissociates from the machinery in the four KOs.

### ALS- or SMA-causative mutations disrupt association of the ASC-1 complex with the RNAP II/U1 snRNP machinery

**Figure 6.**
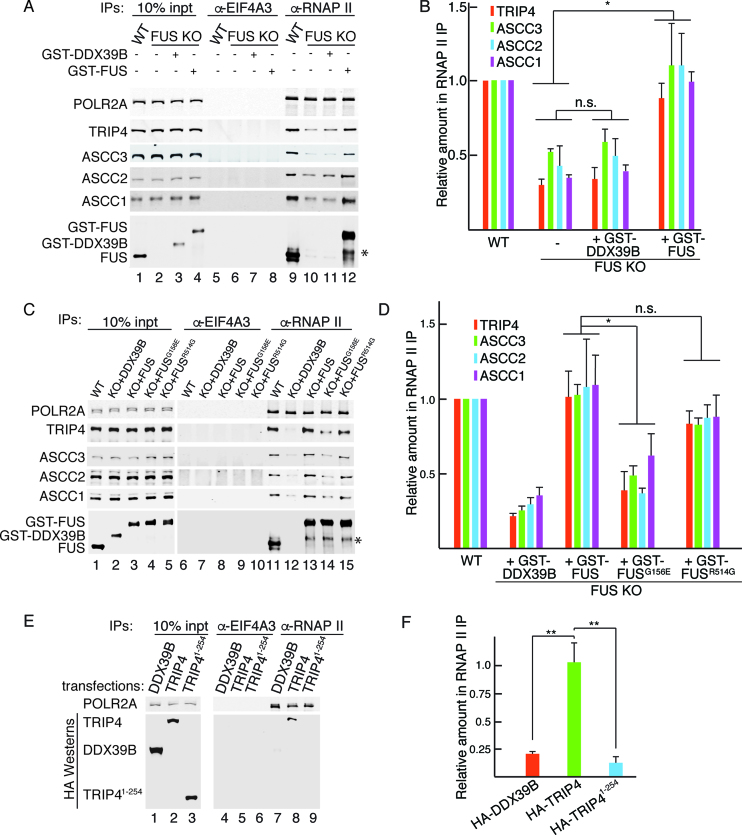
Disease-causing mutations in FUS or TRIP4 disrupt interactions of ASC-1 complex components with the RNAP II/U1 snRNP machinery. (**A**) Purified recombinant GST-FUS or GST-DDX39B was added to FUS KO extract, and RNAP II was IP’d followed by Westerns with antibodies to the ASC-1 components. The asterisk indicates degradation products of GST-FUS. Endogenous FUS, GST-FUS, and GST-DDX39B were detected by the FUS antibody, as this antibody recognizes GST-DDX39B due to the GST tag common to both proteins. (**B**) Three independent replicates of the data shown in (A) were quantitated. The colored bars in the graph show the mean values of fold change for the indicated proteins. Error bars represent standard deviations. *P < 0.05, n.s., not significant (two-tailed Student's *t*-test). (**C** and **D**), same as (A and B), except that GST-FUS^G156E^ or FUSR^514G^ was used for add-backs. (**E**) HA-DDX39B, HA-TRIP4 or HA-TRIP4^1-254^ was expressed in HeLa cells followed by IP/westerns with the indicated antibodies. (**F**) Quantitation of three independent replicates of data shown in (E). **P < 0.01 (two-tailed Student's *t*-test.)

To investigate whether dissociation of the ASC-1 complex from the RNAP II/U1 snRNP machinery could be involved in ALS pathogenesis, we first asked whether WT FUS could restore this association. To do this, equal amounts of purified GST-FUS or a negative control protein (GST-DDX39B) was added back to the FUS KO extract, using levels similar to endogenous FUS (Figure 6A). The RNAP II/U1 snRNP machinery was then immunopurified followed by Westerns with antibodies to the four ASC-1 complex components. As shown in Figure 6A and quantitated in Figure 6B, adding back GST-FUS restored association of the ASC-1 components with the RNAP II/U1 snRNP machinery (lane 12) whereas adding back GST-DDX39B did not (lane 11). This result indicates that FUS is required for association of the ASC-1 complex with the RNAP II/U1 snRNP machinery.

We next used GST-FUS bearing ALS mutations for the add back. FUS^G156E^, which is a mutation in the low-complexity domain and associated with a severe form of ALS, was used for this analysis. In addition, FUS^R514G^, which is a mutation in the nuclear localization signal (NLS) and associated with a less severe form of ALS was also tested. GST-DDX39B and GST-FUS were used as negative and positive controls, respectively. As expected, add back of GST-DDX39B did not restore the association of the ASC-1 complex with the RNAP II/U1 snRNP machinery whereas GST-FUS did (Figure 6C, lanes 12 and 13, and quantitated in Figure 6D). In the case of the mutants, add back of GST-FUS^R514G^, but not GST-FUS^G156E^, restored the association (Figure 6C, lanes 14 and 15, and quantitated in Figure 6D). These data showing that an ALS-causative mutation in FUS disrupts association of the ASC-1 complex with the RNAP II/U1 snRNP machinery indicates a link between ALS and the ASC-1 complex. It is not surprising that the FUS NLS mutation did not have an effect in the *in vitro* add back, as this mutation affects the nucleocytoplasmic localization of FUS in cells. We note that adding GST-FUS^G156E^ to the WT extract did not affect the association of the ASC-1 complex with the RNAP II/U1 snRNP machinery (Supplementary Figure S7), possibly because the mutant protein was not efficiently incorporated into the RNAP II/U1 snRNP machinery due to the stable association of endogenous FUS with this machinery.

We next tested whether an SMA-causative mutation in a component of the ASC-1 complex, TRIP4, affected its association with the RNAP II/U1 snRNP machinery. Because we do not have a KO line of ASC-1 complex components for *in vitro* studies, we carried out a transfection assay. HA-tagged constructs encoding WT TRIP4 (581 aa), an SMA-causative truncated TRIP4 (TRIP4^1-254^) or DDX39B as a negative control were transiently transfected into HeLa cells followed by RNAP II IPs from whole cell lysates. As shown in Figure 6E, all of the HA-tagged proteins were expressed at similar levels (lanes 1–3). However, only WT HA-TRIP4 co-IP’d with RNAP II whereas mutant HA-TRIP4^1–254^ and HA-DDX39 did not (Figure 6E, lanes 7–9, and quantitated in Figure 6F). Thus, an SMA-causative mutation in TRIP4 disrupts its association with the RNAP II/U1 snRNP machinery. Together with our studies of mutant FUS, the results with mutant TRIP4 raise the possibility that dissociation of the ASC-1 complex from the RNAP II/U1 machinery underlies SMA/ALS pathogenesis.

## DISCUSSION

This study together with our previous work (24) revealed that the RNAP II/U1 snRNP machinery is a central hub for ALS- and SMA-causative RNA/DNA binding proteins. For ALS, these proteins are FUS, TAF15, EWSR1, MATR3, TIA1, Valosin Containing Protein (VCP), TARDBP, Heterogeneous Nuclear Ribonucleoprotein A1 (HNRNPA1), and Heterogeneous Nuclear Ribonucleoprotein A2/B1 (HNRNPA2B1), and for SMA, they are SMN1, EXOSC8, HSPB1, TRIP4 and ASCC1. Our observation that such a large number of proteins involved in these motor neuron diseases is present in the RNAP II/U1 snRNP machinery suggests that pathways in which this machinery functions could underlie pathogenesis of these diseases. Another important conclusion from our analysis relates to the multiple functions previously reported for RNAP II and U1 snRNP. In addition to general transcription and splicing, these functions include DNA repair, capping, transcriptional regulation, splicing regulation, and telescripting. The latter process functions to suppress premature cleavage and polyadenylation via U1 snRNP binding to cryptic 5′ splice sites within introns (63). Our data revealing that the RNAP II/U1 snRNP machinery contains multiple complexes with roles in these processes likely explains how this machinery functions in so many processes other than transcription and splicing.

### Dissociation of the ASC-1 complex from the RNAP II/U1 snRNP machinery

Previous work showed that mutations in two (TRIP4 and ASCC1) of the four components of the ASC-1 transcriptional co-activator complex cause a severe form of SMA (32,33). Our work now brings to light the potential importance of the ASC-1 complex to ALS. Specifically, we found that interaction of the ASC-1 complex with the RNAP II/U1 snRNP machinery is disrupted by KO of any of the four ALS-causative proteins examined in our study (FUS, TAF15, EWSR1, MATR3). These data indicate that ALS-causative proteins are required for association of the SMA-causative ASC-1 complex with the RNAP II/U1 snRNP machinery. Importantly, adding back purified GST-FUS, but not GST-FUS bearing an ALS-causative mutation in the low-complexity domain, restored this association, indicating that it may play a role in ALS pathogenesis. Furthermore, our data reveal the ASC-1 complex components themselves as potential new candidates for ALS-causative proteins. Previously, we found that FUS interacts directly with SMN1, providing the first evidence that ALS and SMA are linked at the molecular level. Our new observations regarding the ASC-1 complex indicate that ALS and SMA are more extensively linked at the molecular level than previously thought. In addition, the ASC-1 complex is the first factor identified that is affected in both diseases. The link between ALS and SMA is supported by the observation that both diseases share clinical and neuropathological features (38) and by studies showing that both diseases co-occur in the same family (64).

At present, little is known about the ASC-1 complex. Previous studies showed that knock down of TRIP4 or ASCC1 resulted in severe impairment of neuronal development, which may explain the relationship between the ASC-1 complex and motor neuron disease (32). The ASC-1 complex is a transcriptional co-activator for NF-κB and AP1, both of which have been tied to ALS (31,65–68). Similar to other co-activators, ASC-1 components contain RNA binding motifs and function in splicing (69). Thus, it is possible that dissociation of the ASC-1 complex from the RNAP II/U1 snRNP machinery disrupts transcription and/or splicing of factors involved in ALS and SMA pathogenesis.

A notable difference between ALS and SMA is their mode of inheritance. SMA is usually autosomal recessive whereas ALS is typically autosomal dominant (70,71). It is possible that this difference is related to the age of disease onset. For example, loss of both alleles may be so severe that SMA has an early onset whereas a defect in one allele may lead to the later onset associated with ALS. In this sense, it is possible that SMA and ALS are on two sides of a disease continuum. This possibility is consistent with the observation that the two diseases are linked at the molecular level (38). With respect to the present study, previous work showed that recessive loss of function in ASC-1 components leads to a severe pre-natal form of SMA (32). In contrast, our work suggests that dissociation of the ASC-1 complex from the RNAP II/U1 snRNP machinery due to mutant FUS is ALS causative, which may explain the late disease onset. Further investigation is required to understand how the underlying mechanisms of the different modes of inheritance results in the diseases.

### The RNAP II/U1 snRNP machinery and the DNA damage response

Most of the ALS/SMA proteins have multiple functions, but the precise role(s) involved in disease is not known. The observation that the RNAP II/U1 snRNP machinery is a central hub for so many motor neuron disease-causative proteins raises the question of how this machinery relates to pathogenesis. Among the ALS/SMA proteins present in this machinery, FUS (72), TARDBP (73), TAF15 (74), EWSR1 (75), MATR3 (76), VCP (77), HNRNPA1 (78), HNRNPA2B1(78), SMN1 (51) and HSPB1 (79) all play roles in the DNA damage response (DDR). RNAP II itself also plays a critical role in DDR by sensing DNA lesions during transcription (80,81). Moreover, the RNAP II/U1 snRNP machinery contains a large number of transcription/splicing proteins, such as hnRNPs, that play roles in DDR (82). Finally, this machinery contains multiple complexes that function in DDR. These include the tRNA ligase complex (83,84), NELF (85), P-TEFb (86) and TFIIF (87), and importantly, these complexes dissociate from the RNAP II/U1 snRNP machinery in the different ALS protein KOs. Thus, a possible cause of ALS/SMA pathogenesis is disruption of DDR due to loss of interactions between these DDR complexes/proteins from the RNAP II/U1 snRNP machinery.

Neurons are known to be extremely metabolically active, producing high levels of DNA damage-causing reactive oxygen species (88,89). Neurons are also post-mitotic, lacking the homologous recombination pathway of DDR, which results in accumulation of DNA damage (90,91). Thus, together our data raise the possibility that mutations in ALS proteins dissociate complexes involved in DDR from the RNAP II/U1 snRNP machinery and, over time, DNA damage accumulates in neurons. This mechanism may contribute to neurodegeneration and to the late onset of ALS.

### Distinct roles for the FET proteins

Despite the structural similarity of the FET family proteins (FUS, EWSR1 and TAF15), we found that FUS, EWSR1 and TAF15 are each uniquely required for association of the RNAP II/U1 snRNP machinery with splicing-related factors (U1 snRNP and SMN complex), the tRNA ligase complex, and transcription factors (P-TEFb, TFIIF), respectively. Previous studies reported that the FET family members largely have distinct roles in transcription and/or splicing but the basis for these different functions was not known (92). Our observations regarding the FET family members and the RNAP II/U1 snRNP machinery provide a possible explanation for the distinct roles of these proteins in transcription/splicing.

## Supplementary Material

Supplementary Data
