# Supplementary material for: The neurodegenerative diseases ALS and SMA are linked at the molecular level via the ASC-1 complex
Source: Nucleic Acids Res. 2018 Nov 6;46(22):11939–51. doi: 10.1093/nar/gky1093 (PMC6294556; doi:10.1093/nar/gky1093)
Supplement: Supplementary Data [file gky1093_supplemental_files.zip › Supplementary Information.pdf]

Supplementary Material and Methods

Antibodies

The rabbit polyclonal antibodies to NELFA (cat # 10456-1-AP), NELFB (cat # 16418-1-AP), NELFCD (cat # 11226-1-AP), NELFE (cat # 10705-1-AP), RTCB (cat # 19809-1-AP), DDX1 (cat # 11357-1-AP), FAM98B (cat # 22251-1-AP), RTRAF (cat # 19848-1-AP) and CCNT1 (cat # 20992-1-AP) were purchased from Proteintech (Rosemont, IL). The rabbit polyclonal antibody to CDK9 (cat # A303-493A) was purchased from Bethyl (Montgomery, TX).

IPs from gel filtration fractions

Nuclear extract was separated on a Sephacryl S-500 (GE Healthcare, Marlborough, MA) gel filtration column. A 2.5 mL splicing reaction containing 750 μL of HeLa nuclear extract, 500 μM ATP, 3.2 mM $MgCl_2$ and 20 mM creatine phosphate (di-Tris salt) were incubated for 20 min at 30°C before loading to the column. The gel filtration column buffer contained 20 mM HEPES, 60 mM KCl, 2.5 mM EDTA and 0.1% Triton X-100. Each fraction is 1mL. For IPs, fractions #41 to 57 were combined together. The IP mixture containing 40 μL of antibody crosslinked protein A beads, 0.2 mM PMSF, protease inhibitor EDTA-free (Roche) and 1.5 mL of combined gel filtration fractions was rotated overnight at 4°C, followed by five washes with buffer A. Proteins elution was carried out with the same methods used for IPs from nuclear extract.

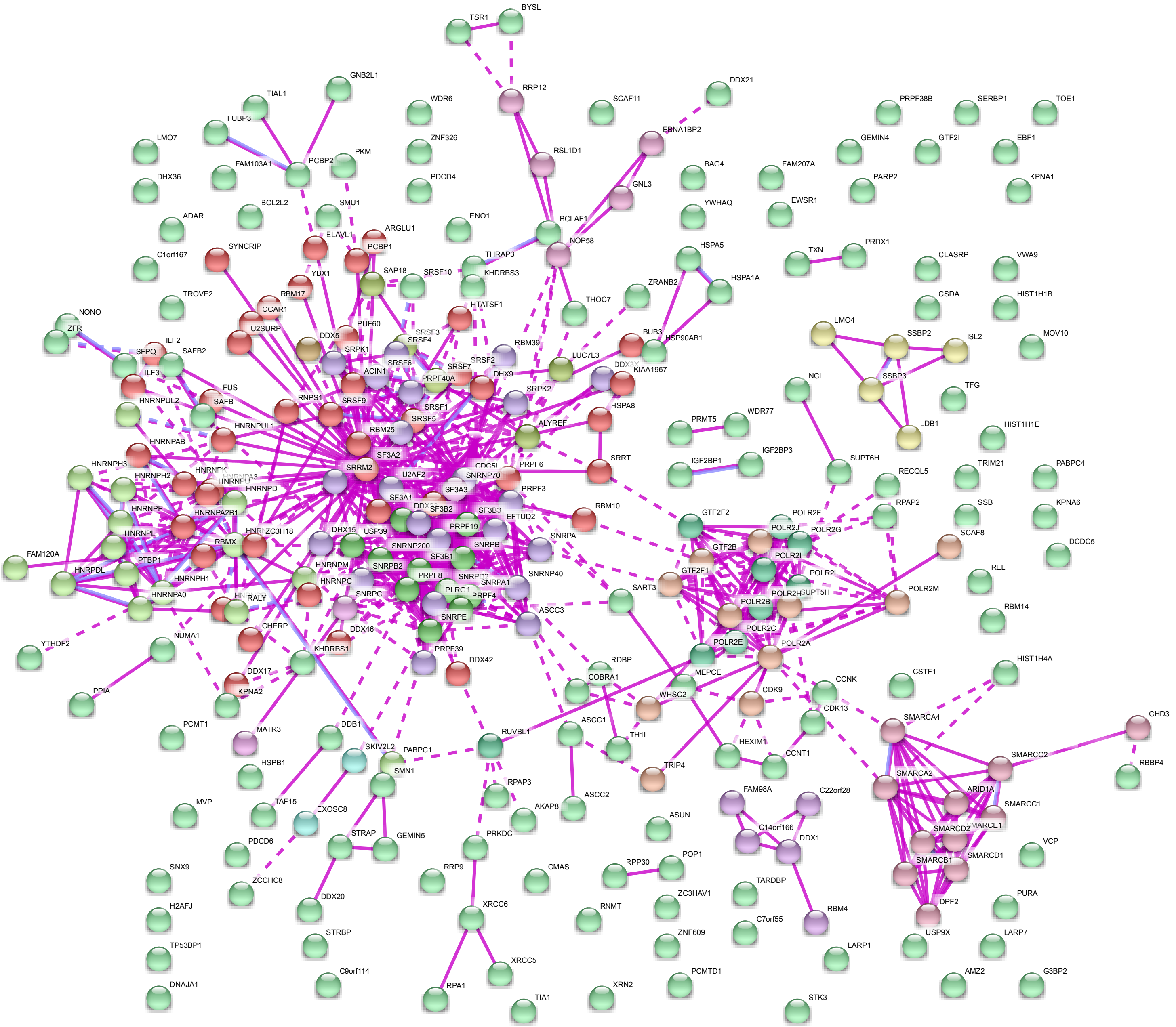

Figure S1. Complete protein interaction network of the RNAP II/U1 snRNP machinery. The original protein interaction network of the RNAP II/U1 snRNP machinery built by the SRTING database (confidence > 0.7, based on experimental data) is shown. The list of proteins in the network is shown in Table S1.

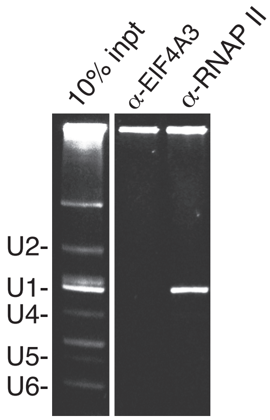

Figure S2. RNAP II specifically co-IP with U1 snRNA. IPs were carried out with the control (EIF4A3) or RNAP II antibody from HeLa nuclear extract. Total RNAs extracted from the IPs were examined on an 8% denaturing gel stained with ethidium bromide. One-tenth of the amount of nuclear extract used for the IP was used as input. The spliceosomal snRNAs are indicated on the left.

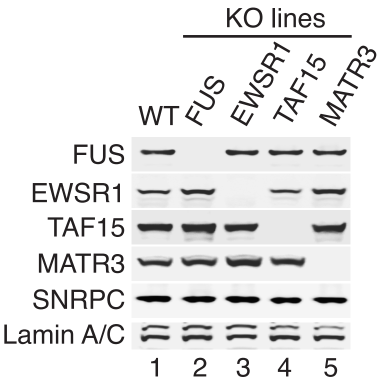

Figure S3. CRISPR knock out of ALS proteins in HeLa cells. Whole cell lysates of wild type (WT) HeLa cells or ALS protein-KO lines were used for westerns with the indicated antibodies. Lamin A/C was used as a loading control.

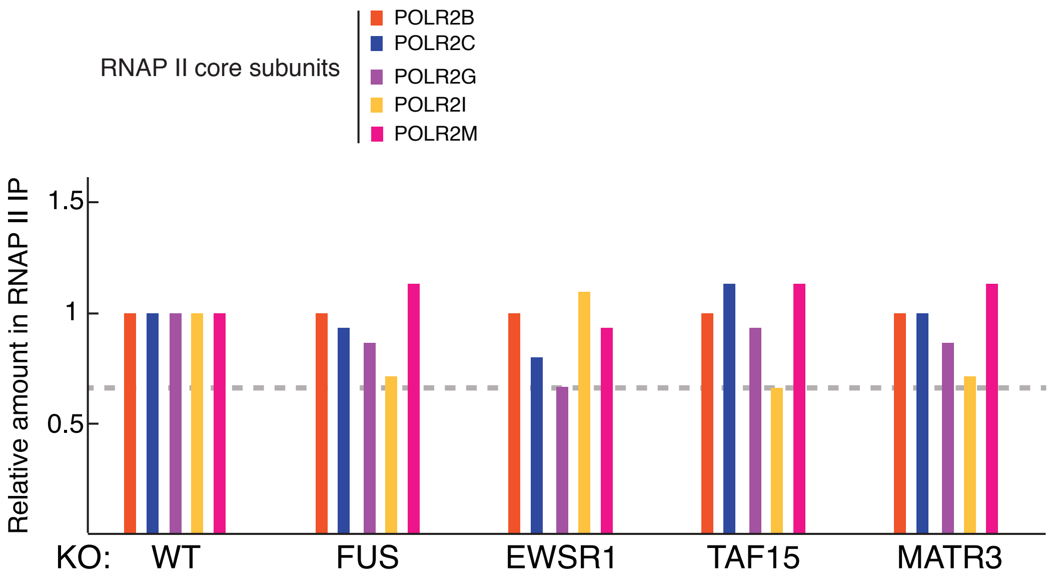

Figure S4. Knockout of ALS proteins does not disrupt association of the core subunits of RNAP II. RNAP II IPs were carried out from wild type or indicated knockout (KO) nuclear extracts and analyzed by quantitative mass spectrometry. Bar chart of the relative amounts of the RNAP II core subunitsin the RNAP II IP from each extract is shown. The dashed line indicates a fold change of -1.5.

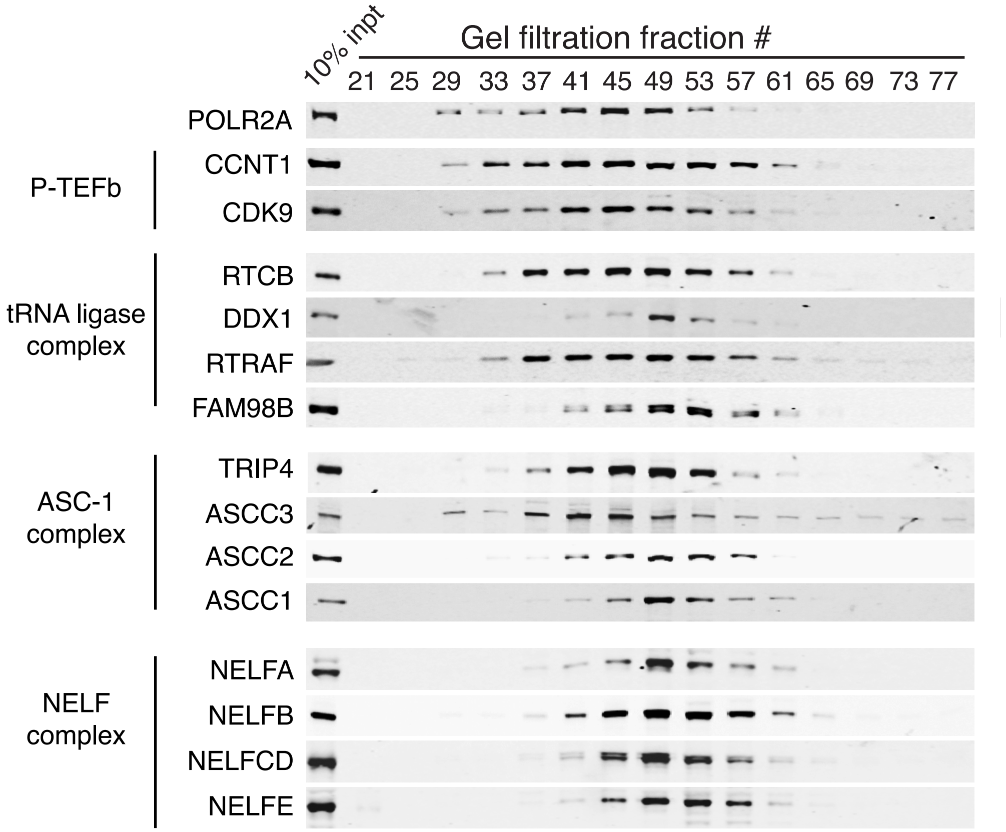

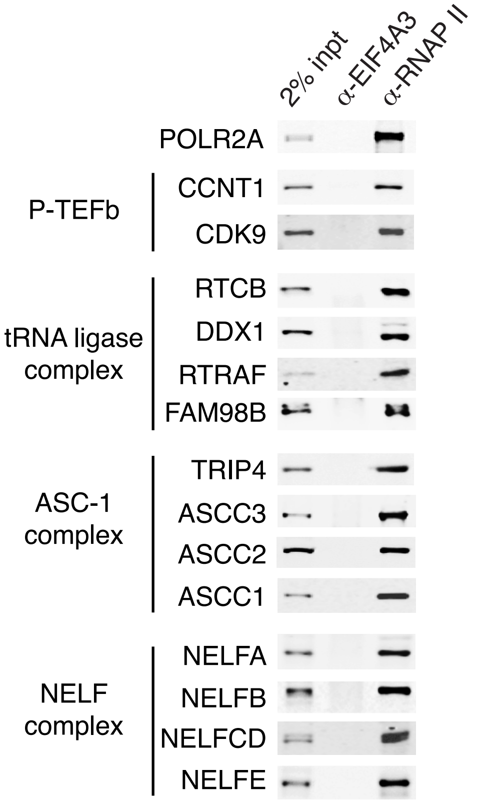

Figure S5. Validation of complexes identified in the RNAP II/U1 snRNP machinery. (A) HeLa nuclear extract was seperated on a Sephacryl S-500 column. Every fourth fraction was used for Westerns with antibodies to the components of the indicated complexes. (B) RNAP II IP was carried out from fractions #41 - 57 followed by Western analyses using indicated antibodies.

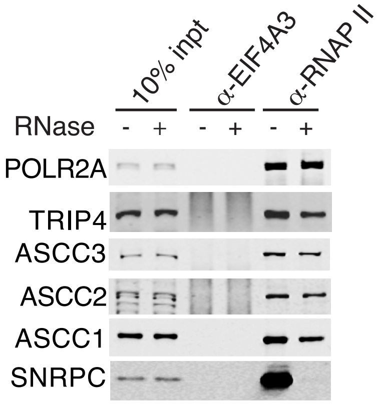

Figure S6. The ASC-1 complex associates with the RNAP II/U1 snRNP machinery in an RNA-independent manner. (A) IPs were carried out from RNase A-treated or untreated nuclear extract using an RNAP II or negative control antibody (EIF4A3) followed by Westerns with indicated antibodies.

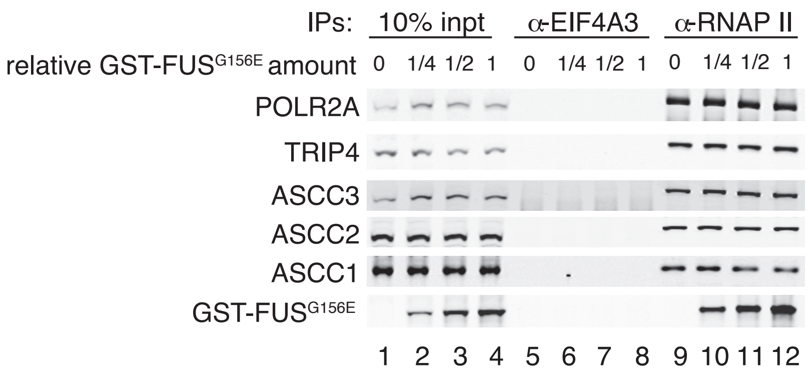

Figure S7. Adding mutant FUS protein to the WT extract does not affect the association of the ASC-1 complex with the RNAP II/U1 snRNP machinery. Different amounts of purified recombinant GST-FUS^{G156E} were added to WT extracts, and IPs were carried out using an RNAP II or negative control antibody (EIF4A3) followed by Westerns with indicated antibodies. The amounts of GST-FUS^{G156E} proteins relative to the amount of endogenous FUS were indicated.
